# Pilot optimization trial of a sexual and reproductive health program for Latina teens and their female caregivers: A study protocol

**DOI:** 10.1016/j.conctc.2025.101512

**Published:** 2025-06-23

**Authors:** Katherine G. Merrill, Jacqueline Silva, Wendy Chu, Gisel Romero, Vanessa Melgoza, Blanca Gabino, Corin Mora, Sara Vargas, Jacqueline Fuentes, Caitlin Kelleher-Montero, Nicholle Courrejolles, Kate Guastaferro, Felicia Scott-Wellington, Susana Salgado, Angela Sedeño

**Affiliations:** a Center for Dissemination and Implementation Science, University of Illinois Chicago, United States; b Floreciendo Community Advisory Council, United States; c Department of Psychiatry, University of Illinois Chicago, United States; d Department of Psychology, University of South Carolina, United States; e LoSAH Center of Hope, United States; f Corazón Community Services, United States; g Gads Hill Center, United States; h Centro Romero, United States; i The Center for Relational Wellness, United States; j Feinberg School of Medicine, Northwestern University, United States; k Department of Medicine, University of Illinois Chicago, United States; l School of Global Public Health, New York University, United States; m Department of Pediatrics, University of Illinois Chicago, United States; n Expanded Mental Health Services of Chicago NFP, United States

## Abstract

**Background:**

Latina teens experience sexual and reproductive health disparities; however, few effective interventions designed for Latina teens and their families exist. Floreciendo is a sexual and reproductive health intervention for Latina teens (14–18 years) and their female caregivers (e.g., mothers, sisters), delivered by trained staff at community partner organizations (CPOs).

**Methods:**

This protocol describes a hybrid type 2 mixed-methods study with a pilot 2^3^ factorial experimental design which draws on the multiphase optimization strategy (MOST) framework. Small groups of teen-caregiver dyads (target n = 92 dyads/184 participants) will be randomized to 1 of 8 conditions across four CPOs. All will receive the Foundations in Sexual Risk Prevention (i.e., constant) component. Groups of dyads will be randomized to different combinations of three intervention components of Floreciendo, which are either “on” or “off”: 1) Condoms and Contraception, 2) Family Strengthening, and 3) Gender and Relationships. Our aim is to examine the feasibility of using a factorial design and the acceptability of the intervention components. We will also explore effectiveness outcomes—including risky sexual behavior (primary) and incidence of sexually transmitted infections and unplanned pregnancy (secondary)—and implementation outcomes, including appropriateness, feasibility, adoption, sustainability, cost, and fidelity. Qualitative data will build on quantitative data. We will conduct focus group discussions and key informant interviews with Latina teens, female caregivers, facilitators, CPO leadership, and collaborators.

**Discussion:**

Results will be used to guide intervention component and implementation refinement and will inform plans to conduct a fully powered optimization trial of Floreciendo.

## Highlights

• Latina teens face sexual and reproductive health disparities.
• Floreciendo is a Latina teen-female caregiver sexual & reproductive health program.
• This protocol details the Floreciendo pilot optimization trial.

## 1 Background

Latina teens face a disproportionate burden of STIs, including HIV, and other adverse effects of risky sexual behavior. Compared to their White counterparts, Latina teens have higher rates of syphilis, chlamydia, gonorrhea, and HIV/AIDS [1], lower rates of effective birth control at last sex [2], and more than double the rate of teen pregnancy [3]. Latina teens also have high rates of mental health challenges [4], intimate partner violence (IPV) [5,6], and risky substance use [7,8]. These health disparities are influenced by unique contextual factors facing Latina teens and their families, including acculturation, language barriers, immigrant status, religion, and cultural norms (e.g., machismo) [9].

Parents influence the sexual behavior of their teens, and positive parenting practices (e.g., monitoring, open parent-child communication) can deter teens' risky sexual behaviors [10]. More frequent mother-daughter communication about sex is associated with teens' improved beliefs in taking responsibility for contraceptives, as well as confidence in and frequency of communicating with partners [[11], [12], [13]]. Sexual health interventions for Latine teens and their family members have shown positive effects on parent-teen communication and teens’ sexual behavior [[12], [13], [14], [15], [16]]. However, a scoping review conducted by our team identified no existing evidence-based sexual and reproductive health interventions for Latina teens and their female caregivers specifically (publication forthcoming).

Floreciendo is a sexual and reproductive health intervention for Latina teens and their female caregivers comprised of four 2-h sessions. Over two years, our team adapted Floreciendo [[14], [17], [18]] from IMARA, an evidence-based mother-daughter intervention for Black families [15,16]. While a traditional approach would involve piloting and assessing the intervention as a package [19,20], we were interested in unpacking the “black box” of Floreciendo. Specifically, we wished to assess which of its intervention components impact teens’ sexual and reproductive health outcomes, independently and in combination with each other. Our approach was shaped by the multiphase optimization strategy (MOST) framework, which guides the development and optimization of a multi-component intervention that produces the best expected outcome within key constraints imposed by the need for efficiency, affordability, and/or scalability [19,20]. The MOST framework includes three phases focused on preparing, optimizing, and evaluating the intervention. Piloting the intervention and the experimental design selected for the eventual optimization trial is a critical activity in the preparation phase [19,20].

This study protocol describes a pilot optimization trial of Floreciendo. Reflecting the purpose of the preparation phase in MOST, our aim is to examine the feasibility of using a factorial design and acceptability of the intervention components to inform the subsequent optimization and evaluation phases via a fully powered optimization trial. We also seek to explore effectiveness outcomes, implementation outcomes, mediators, and proximal behavioral outcomes to inform intervention component refinement and future implementation. This study uses a community-based participatory research (CBPR) approach [21], guided by the Floreciendo Community Advisory Council, made up of representatives from community partner organizations (CPO), researchers, Latina teens and female caregivers, and other experts in Latina health. For this paper, we drew on the SPIRIT guidelines for reporting on clinical trials [22] and guidelines for reporting on preparation phase activities of the MOST framework [23].

## 2 Methods

### 2.1 Study design

This is a hybrid type 2 effectiveness – implementation mixed methods study and uses a 2 × 2 × 2 (or 2^3^) factorial experimental design for the pilot optimization trial. We chose a hybrid type 2 design given that we aim to run a fully powered optimization trial with equal interest in effectiveness and implementation outcomes [24] and given that we adapted Floreciendo from an evidence-based program [15,16]. We chose to pilot a factorial trial design given our ultimate goal of optimizing the Floreciendo intervention using the MOST framework [19]. We decided on a mixed methods approach to facilitate a deeper understanding of exploratory effectiveness and implementation outcomes than would be possible with either method alone [25].

**Fig. 1:**
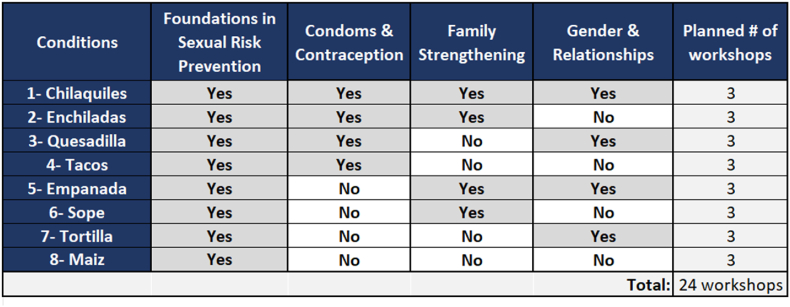
Conditions and planned number of Floreciendo workshops.

All dyads of Latina teens and their female caregivers receive the first of the four intervention sessions of Floreciendo (i.e., the “constant,” Foundations in Sexual Risk Prevention). Dyads are randomized to receive different combinations of the remaining three sessions which are either delivered (i.e., “on”) or not delivered (i.e., “off”): Condoms and Contraception, Family Strengthening, and Gender and Relationships. The study design therefore includes eight experimental conditions. Sessions are led by trained staff at each CPO. To facilitate easier communication about the conditions and enhance adherence to condition delivery [26], our Community Advisory Council chose to name each condition after a popular food in Latine culture, on a scale from most complex (i.e., chilaquiles) to simplest (i.e., maiz) (Fig. 1). In using a factorial design, there is no typical control condition; that is, participants are used in the estimation of all main effects and interactions [27].

We plan to enroll about 92 dyads (ñ184 participants total). Floreciendo is designed to be delivered in small groups of roughly 3–5 dyads. To achieve our planned sample size, we expect to deliver about 24 workshops. Our optimization objective [20] is to arrive at the best expected outcome among all active conditions (i.e., with no time or cost restrictions on the number of factors which can be “on”). This objective was determined through our formative Floreciendo research, whereby no specific constraints (i.e., limitations on cost or time) for delivering the intervention were raised by Latina teens, female caregivers, or CPO staff/leadership [17]. We will collect qualitative data after collecting quantitative data, with greater emphasis placed on the quantitative data (i.e., using a QUANT → qual design) [25].

### 2.2 Setting and partners

The study setting is the Chicagoland area, where Latines represent the second largest racial/ethnic group and comprise one-third of the region's population [28]. This study is being conducted through a partnership between the Center for Dissemination and Implementation Science at the University of Illinois Chicago (UIC) and four CPOs: 1) Expanded Mental Health Services of Chicago (EMHS), which includes two community-funded mental health centers (The Kedzie Center and the LoSAH Center of Hope); 2) Centro Romero, a social service organization serving Latine refugee and immigrant communities on the North side of Chicago; 3) Corazon Community Services, a social service organization serving Latine families in Cicero, Illinois; and 4) The Gads Hill Center, a social service organization serving children and their families on the West side of Chicago. The Floreciendo Community Advisory Council, launched at study conception [14], meets bi-monthly and provides guidance on all aspects of the study.

### 2.3 The floreciendo intervention and its components

Floreciendo, meaning “blooming” in Spanish, is a sexual and reproductive health intervention designed for Latina teens (aged 14–18 years old) and their female caregivers (e.g., mothers, sisters, aunts, grandmothers) generated using systematic process based on existing literature [14]. It aims to reduce risky sexual behavior to prevent STIs and unplanned pregnancy, while promoting mental health and reducing IPV and risky substance use. In line with the IMARA program, Floreciendo draws on the social personal framework [29], recognizing multiple levels of influence on teens’ sexual and reproductive health from individual, social, and environmental factors.

In interactive workshops conducted in-person at CPOs, teens and caregivers engage in joint and separate activities. All sessions begin with joint introductory activities in the same space. Then, dyads are separated for teens and caregivers to learn parallel content without judgment. Finally, dyads return to the same space for joint closing activities. Participants indicate their preferred language (English or Spanish) at the beginning of the first session. If all teens and/or caregivers agree on a language, that language is used throughout. If there are different levels of comfort with language, simultaneous translation is used. The workshop emphasizes strong teen-caregiver relationships and seeks to build motivation for protection from sexual health risks, while strengthening caregivers’ credibility as a resource for reducing sexual risk behavior.

Floreciendo is made up of four 2-h sessions, each addressing a topic believed to support sexual and reproductive health decision-making:

• *Foundations in Sexual Risk Prevention:* Covers key topics in sexual and reproductive health and helps teens and caregivers develop an awareness of sexual behavior risks.
• *Condoms and Contraception:* Teaches different forms of condoms and contraception and offers experiential activities on using them.
• *Family Strengthening:* Facilitates learning and practice in how teens and caregivers can communicate about sex and addresses family norms and expectations in Latine culture.
• *Gender and Relationships*: Shares what a healthy relationship is and how to recognize IPV and discusses how gender and cultural norms in Latine culture influence teens' sexual behavior.

### 2.4 Conceptual model

**Fig. 2:**
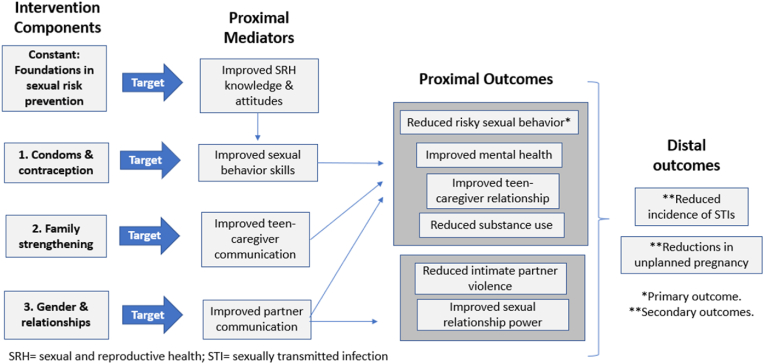
Conceptual model depicting links between intervention components and desired outcomes.

Our conceptual model depicts the links between the intervention components and reductions in risky sexual behavior (primary distal outcome) and STI incidence and unplanned pregnancy (secondary distal outcome). Each component addresses one proximal mediator, which is linked to the desired distal outcomes through one or more proximal behavioral outcome (Fig. 2).

### 2.5 Eligibility criteria

We are enrolling Latina teens and their female caregivers, defined as an influential adult female in the teen's life (e.g., mother, sister). To be eligible, teens must identify as Latina and be 14–18 years old. Caregivers must be a female caregiver of a Latina teen aged 14–18 years old and be 19 years old or older. Teens and caregivers must speak English and/or Spanish, be living with or in daily contact with each other, be available to simultaneously participate in the workshop and research activities, and agree to participate as a dyad. Teens and caregivers are excluded if they are unable to provide informed consent/assent or if they participated in the theater testing that informed this pilot optimization trial [18].

### 2.6 Randomization and masking

Given the large number of conditions [30], the randomization schedule is generated prior to the study start by the study statistician and organized in Excel. Groups of dyads are randomly assigned to 1 of 8 conditions (Fig. 1) in blocks of 8. Conditions are revealed line by line as the recruitment of each group of dyads occurs. The Floreciendo Project Director manages the randomization schedule and communicates assignments to CPO sites. Each CPO has an equal chance of delivering any condition, and each intervention component will be delivered a roughly equal number of times by the study end. Facilitators at each CPO are trained in delivering all intervention components given the relatively small sample size, as it would not be feasible for each CPO to deliver all eight conditions.

CPO and UIC staff are blinded to the condition when approaching and recruiting participants. Only key UIC staff, facilitators, and CPO supervisors are aware of the condition for the purposes of delivering the workshop and are informed not to disclose the condition to participants prior to the workshop. Teens and caregivers are informed of their condition just before the workshop starts and are asked not to discuss their condition assignment with anyone outside of their group. To measure possible contamination, participants are asked on follow-up surveys the extent to which they discussed content received in their condition with participants randomized to other conditions.

### 2.7 Procedures

**Fig. 3:**
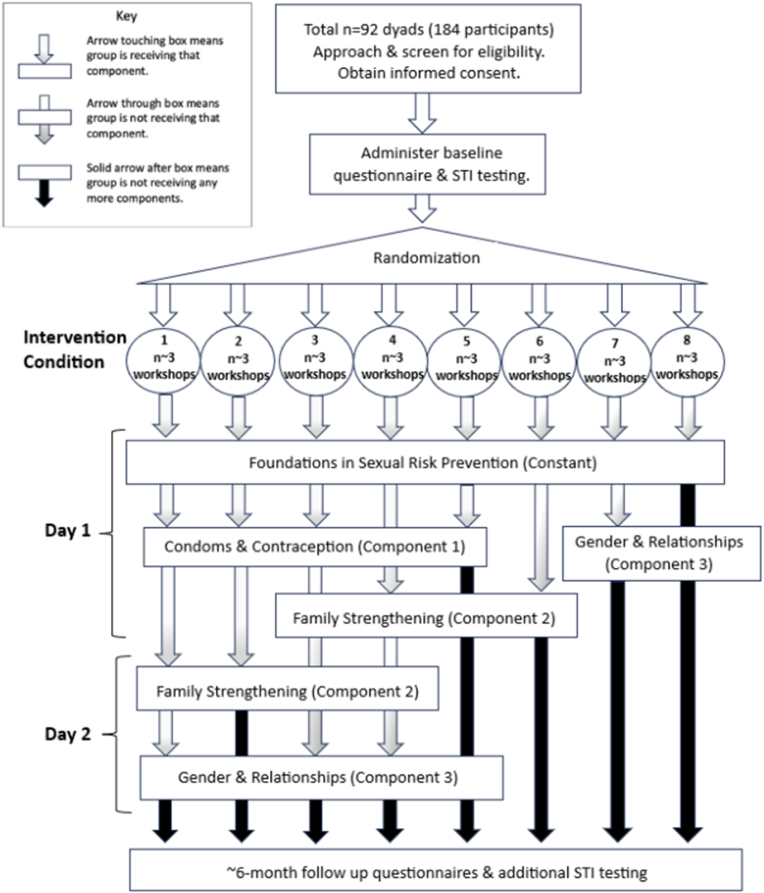
Schema for the pilot optimization trial of Floreciendo.

CPO staff and/or UIC study staff approach potential participants using an interest form which collects contact information. UIC study staff recruit potential participants by confirming eligibility, gathering informed consent, and registering them for a workshop. Groups of participants are randomly assigned to complete 1 of 8 intervention workshop conditions at a CPO site for 1 or 2 days (Fig. 3). All receive the constant component. Groups randomized to receive one additional component attend a one-day workshop, and groups randomized to receive two or more additional components attend a two-day workshop (see conditions in Fig. 1). Each participant receives between $100 and $220 depending on the condition they are randomized to and whether selected for a focus group discussion. Compensation includes $25 for each workshop session attended; our formative research highlighted the importance of compensating participants for their time attending sessions, not just completing assessments [17]. Compensation also includes $5 to cover transport for study activities [17].

Prior to the workshop start, teens and caregivers complete a baseline questionnaire. Teens also provide a urine sample to be tested for three common STIs: gonorrhea, chlamydia, and trichomoniasis. Urine samples are tested in a lab using a PCR test, and teens are called with their results when they are received by the study team (typically within 2–3 business days). Immediately after each workshop session, participants complete a brief feedback survey on the session's acceptability. After finishing all sessions per their condition, participants complete a comprehensive feedback survey about the acceptability, appropriateness, and feasibility of the sessions. Breakfast, lunch, and childcare are provided at the workshop, based on feedback received during formative research [17]. Approximately 6 months later, caregivers and teens are asked to complete a follow-up questionnaire, and teens are asked to provide another urine sample for STI testing. While questionnaires can be completed remotely, STI testing for teens is done in person and uses the same procedures as at baseline.

Teens who test positive for an STI at baseline or follow-up are offered treatment for themselves and their partner from an adolescent medicine physician, paid for by the study. In line with the Centers for Disease Control's recommended regimen for gonorrhea and alternative regimens for chlamydia and trichomoniasis [31], single treatment regimens with directly observed therapy are offered to increase medication adherence. Alternative regimens are used if participants have allergies. When providing treatment, the adolescent medicine physician counsels teen participants on ways to prevent reinfection. The physician also recommends that the participant follow up with their primary care provider or offers recommendations of providers to establish care.

Floreciendo facilitators are invited to complete surveys after receiving facilitator training (described below) to provide feedback on the training and how it could be improved. Additionally, facilitators, CPO leadership, UIC staff, and Floreciendo collaborators (e.g., Community Advisory Council members) are invited to complete implementation surveys near the time of intervention delivery completion (estimated to be about 18 months after study start) or before staff turnover. These surveys measure the acceptability, appropriateness, and feasibility of the intervention and its implementation. CPO facilitators and leadership surveys also measure intentions to adopt and sustain the intervention.

### 2.8 Facilitator training

Based on formative research informing the current study [14], facilitators delivering Floreciendo must be Latina, fluent in English and/or Spanish, and identified from within the CPOs. Where possible, facilitators are paired with teens or caregivers to facilitate similar age or experience with motherhood. At least one facilitator per workshop is bilingual. Facilitators are trained by UIC staff. Initial training of facilitators lasts approximately 18 h, with two full days of in-person sessions and the remaining sessions occurring remotely. Facilitators who are trained after the study start receive one full day of in-person sessions and remote sessions and are asked to observe a full workshop. Based on best practices [32], trainers follow a standardized training manual and protocol and provide ongoing support and consultation. Training content covers the objectives and content of all sessions, tips in facilitation (e.g., in addressing challenging group dynamics), the importance of fidelity, and how to manage disclosure of suicidality and abuse. Modeling, role-play, and discussions are used to reinforce learning and to build a sense of community among facilitators. As part of their training, facilitators are also required to complete Sex Ed To-Go modules on STIs, pregnancy prevention (including abstinence), healthy relationships, and communication skills [33].

### 2.9 Ethical considerations and safety monitoring

The study protocol has been approved by the Institutional Review Board at the University of Illinois Chicago. Written informed consent is obtained from teens aged 18 years and female caregivers. Written teen assent and parent permission is required for teens who are minors (i.e., ages 14–17 years old). Teens’ refusal supersedes parent permission. The principal investigator oversees potential social harms reported by teens in partnership with leadership staff at CPOs. On surveys, teens are flagged if they report any intimate partner violence in the past year, any thoughts of suicide in the past two weeks, or poor wellbeing. Flagged teens are discreetly escorted to a designated CPO staff member, who is trained to assess harm severity and facilitate safety planning. Disclosures of abuse, violence, or significant harm to self or others are recorded and reported to appropriate authorities. A data and safety monitoring board meets annually to oversee participant safety.

### 2.10 Sample size

As is appropriate for pilot studies, this study aims to understand the feasibility and acceptability of running an optimization trial and thus is not explicitly powered to detect statistically significant effects [34]. However, we will conduct exploratory analyses of intervention component effects on effectiveness outcomes, proximal behavioral outcomes, and theoretical mediators (detailed below).

### 2.11 Outcomes

#### 2.11.1 Primary outcomes

Our primary outcomes are: 1) feasibility of using a factorial design, and 2) acceptability of the intervention components. We will assess the feasibility of using a factorial design by measuring the proportion of conditions implemented according to their randomization schedule (benchmark is ≥ 90 %). We will also examine the proportion of dyads who complete all sessions specified per their condition (benchmark is ≥ 80 %) and the proportion who complete baseline assessments (benchmark is ≥ 80 %) and follow-up assessments (benchmark is ≥ 70 %). We will assess the acceptability of the Floreciendo intervention components—i.e., the extent to which participants perceive the components to be agreeable, palatable, or satisfactory [35]—by measuring the proportion of teens and caregivers who report high satisfaction with the intervention components. We will measure acceptability using items adapted the mHIST [36] (benchmark is ≥ 80 % agreement).

#### 2.11.2 Exploratory effectiveness outcomes

While this pilot study is not powered to estimate effects of the components [34], it provides an opportunity to explore the potential for effectiveness in a future, adequately powered trial. To this end, we will assess teens’ risky sexual behavior by measuring the proportion of teens who report risky sexual behavior at follow-up, self-reported using AIDS Risk Behavior Assessment [37]. We anticipate this will serve as our primary effectiveness outcome in our future optimization trial. Our secondary outcomes in the future optimization trial will include STIs and unplanned pregnancy. In this pilot, we will measure the proportion of teens who screen positive for any of three STIs and the proportion of teens with an unplanned pregnancy (based on self-report) at follow-up. Where sample sizes allow, we will explore mediators and proximal behavioral outcomes in line with our conceptual model, including mental health, teen-caregiver relationships, substance use, IPV exposure, sexual relationship power, and gender equitable norms.

#### 2.11.3 Exploratory implementation outcomes

In addition to examining the acceptability of the intervention components (described above), we will explore other implementation outcomes of Floreciendo, in line with Proctor et al.‘s implementation outcomes framework [35]:

• *Feasibility*: The extent to which Floreciendo can be successfully carried out in this setting (distinct from the feasibility of using a factorial design described above);
• *Appropriateness*: The extent to which participants believe Floreciendo to be relevant or compatible with the setting and Latina community;
• *Adoption*: The proportion of CPOs willing to initiate Floreciendo;
• *Sustainability*: The intention of the CPOs to maintain or institutionalize Floreciendo;
• *Cost*: Costs associated with the implementation of Floreciendo;
• *Fidelity*: The extent to which Floreciendo is implemented as intended.

We will quantitatively measure acceptability, feasibility, and appropriateness of the intervention components (i.e., the intervention content and implementation strategies) among participants (i.e., teens and caregivers) and facilitators/staff/collaborators using items adapted from the mhIST [36]. Among facilitator/staff/collaborators, we will measure adoption using items adapted from the Antiretroviral Treatment Access Study (ARTAS) [38] and sustainability intentions using items adapted from the Program Sustainability Assessment Tool (PSAT) [39]. We will track costs associated with intervention delivery (e.g., personnel costs, workshop supplies) to get an early indication to inform the future optimization trial.

We will assess fidelity by measuring facilitator and observer ratings of adherence [[40], [41], [42]], quality of delivery [[40], [43]], and participant responsiveness [[32], [44]]. Fidelity forms will be reviewed regularly by an experienced trainer on the study team and the CPO site supervisor to provide feedback. Facilitators who are not reaching ≥80 % fidelity will be required to receive additional training.

### 2.12 Qualitative data collection

We aim to conduct about four focus group discussions with Latina teens, four with female caregivers, and two with facilitators, each lasting approximately 90 min. We will recruit about 6–8 participants per focus group. Additionally, we aim to conduct roughly six 30-min key informant interviews with CPO leadership and other collaborators (e.g., Community Advisory Council members). Focus groups and interviews will be hosted in person at a location convenient (e.g., a private room at a CPO) or remotely (e.g., Zoom) using a semi-structured guide. Open-ended questions will build on the quantitative survey questions to explore perceived effects of the workshop on participants and perceptions of implementation outcomes. Focus groups and interviews will be conducted in English or Spanish, digitally recorded, translated as needed, and transcribed.

### 2.13 Retention

At enrollment, we will obtain contact information from participants, including of at least two people who could help us locate participants. We will ask participants for their permission to send them study material and reminders via text messages. If more than three contact attempts fail, we will reach out to the contacts from participants' records, request assistance from the recruiting CPO, visit the family's last known address, and leave letters at the home encouraging call-back. Remuneration for participants' time completing surveys will increase from $30 at baseline to $35 at follow-up to support retention.

### 2.14 Data management

Data management will be housed in REDCap, a password-protected and user-restricted application [45]. Exported participant data, linked by an assigned numerical ID, will be stored in secure servers maintained by UIC. Teen participants are also assigned a medical record number, used to label their urine samples. After screening the urine for STIs, the laboratory adds teen participants’ STI results directly to their electronic medical record using the medical record number; this is how STI test results are shared with the study team and the adolescent medicine physician providing treatment. The principal investigator will review any requests to use the data externally, and data shared will be stripped of identifiers.

### 2.15 Analyses

We will assess the feasibility of using a factorial design and acceptability of intervention components using descriptive statistics. To explore effectiveness outcomes in support of our move towards running a fully powered optimization trial, we will conduct an intention-to-treat analyses. We will use mixed-effects models for continuous outcomes and generalized estimating equations for binary outcomes. We will examine the mean difference in risky sexual behavior scores (primary outcome) for each intervention component from baseline to follow-up. Where sample sizes allow, we will do the same for STI incidence and unplanned pregnancy. Each experimental factor will be coded at two levels using effect coding (“on” = 1; “off” = −1) [19]. We will replicate analyses to look at component effects on theoretical mediators and proximal behavioral outcomes and explore the possibility of examining mediation effects and interactions between components.

For exploratory implementation outcomes, we will conduct descriptive analyses to summarize quantitative measures from participant and facilitator/staff/collaborator implementation surveys, as well as the facilitator and observer fidelity forms. We will use the *planning for and assessing rigor in rapid qualitative analysis (PARRQA)* rapid qualitative analysis framework [46] to analyze open-ended responses, focus group discussion transcripts, and key informant interview transcripts. Data will be sorted using a matrix, with relevant data extracted and key themes refined through extensive discussion among the analysis team and in consultation with the project's Community Advisory Council.

### 2.16 Dissemination

Study findings will be disseminated widely through peer-reviewed publications, conferences, presentations, CPO staff and community meetings at each CPO, and related avenues. We will share findings with study participants through study briefs and invitations to community meetings. The principal investigator will approve all dissemination materials. Data will be uploaded to a repository for public access after study completion.

## 3 Discussion

This pilot study responds to a pressing need among the Latine community to support Latina teens with their sexual and reproductive health [[1], [47]]. While studies have demonstrated the value of parent-teen sexual health interventions [[15], [48], [49]] and of family interventions for Latine teens [50], to our knowledge, there are no existing evidence-based interventions of sexual and reproductive health for Latina teens and their female caregivers. Such interventions are necessary to reduce recognized disparities in sexual and reproductive health outcomes among Latina teens [[1], [2], [3]].

This study has several strengths. First, interventions are typically delivered and tested as a bundled package without an understanding of whether all intervention components are contributing meaningfully towards the achievement of desired outcomes. The MOST framework provides a tool for efficiently testing the effectiveness of the components of Floreciendo prior to testing the intervention as a whole [19,20]. The current pilot optimization trial in the preparation phase of MOST will lay the foundation for a fully powered optimization trial of Floreciendo in the next phase of this work. Second, this study addresses calls in the literature for mother-daughter sexual and reproductive health interventions for Latina teens [[14], [51]]. While our focus is on improving health outcomes among teens, we will engage both teens and their female caregivers to create a culture of support for prevention. Third, from the outset, the Floreciendo project has been carried out using a CBPR approach. Community-engaged research methods are recognized as a key value-add to studies broadly [21] and is a growing area of research within studies using the MOST framework [[52], [53], [54]]. Using a CBPR approach, our team rigorously adapted Floreciendo over two years to respond to the needs of Latina teens [[14], [17], [18]]. Finally, our team planned for adoption and sustainability from the start of the project and made decisions to support their achievement, such as the decision to train staff from CPOs as facilitators and deliver Floreciendo in community settings. This planning will help to position Floreciendo for high adoption and sustainability and potentially for scale [55,56].

## CRediT authorship contribution statement

**Katherine G. Merrill:** Writing – review & editing, Writing – original draft, Visualization, Supervision, Project administration, Methodology, Investigation, Funding acquisition, Conceptualization. **Jacqueline Silva:** Writing – review & editing, Writing – original draft, Visualization, Project administration, Methodology, Investigation, Conceptualization. **Wendy Chu:** Writing – review & editing, Writing – original draft, Project administration, Investigation. **Gisel Romero:** Writing – review & editing, Methodology, Investigation. **Vanessa Melgoza:** Writing – review & editing, Methodology, Investigation. **Blanca Gabino:** Writing – review & editing, Methodology, Investigation. **Corin Mora:** Writing – review & editing, Methodology, Investigation. **Sara Vargas:** Writing – review & editing, Methodology. **Jacqueline Fuentes:** Writing – review & editing, Methodology. **Caitlin Kelleher-Montero:** Writing – review & editing, Writing – original draft, Project administration, Investigation. **Nicholle Courrejolles:** Writing – review & editing, Writing – original draft, Investigation. **Kate Guastaferro:** Writing – review & editing, Methodology. **Felicia Scott-Wellington:** Writing – review & editing, Methodology. **Susana Salgado:** Writing – review & editing, Supervision, Methodology, Investigation, Conceptualization. **Angela Sedeño:** Writing – review & editing, Supervision, Methodology, Conceptualization.

## Protocol registration

NCT06223165.

## Ethics approval and consent to participate

In accordance with the Declaration of Helsinki, written informed consent and parental permission for minors was obtained from all individual participants included in the study. Ethics approval was obtained from the Institutional Review Board at the University of Illinois Chicago (IRB 00000116).

## Consent for publication

Not applicable.

## Availability of data and materials

Not applicable.

## Funding

This research was supported by the 10.13039/100009633Eunice Kennedy Shriver National Institute of Child Health and Human Development (to KM) (R00HD105490). The funders had no role in the design and conduct of the study; collection, management, analysis, and interpretation of the data; preparation, review, or approval of the manuscript; or decision to submit the manuscript for publication.

## Declaration of competing interest

The authors declare that they have no known competing financial interests or personal relationships that could have appeared to influence the work reported in this paper.

## Data Availability

No data was used for the research described in the article.
